# Risk prediction model for severe potential drug-drug interactions in colorectal cancer patients: a real-world data study

**DOI:** 10.3389/fphar.2025.1714838

**Published:** 2026-01-12

**Authors:** Xiaomei Pei, Xiaohu Yang, Lingti Kong

**Affiliations:** 1 Department of Pharmacy, The First Affiliated Hospital of Bengbu Medical University, Bengbu, China; 2 School of Pharmacy, Bengbu Medical University, Bengbu, China; 3 School Hospital, Zaozhuang Vocational College, Zaozhuang, China; 4 Institute of Emergency and Critical Care Medicine, The First Affifiliated Hospital of Bengbu Medical University, Bengbu, China

**Keywords:** colorectal cancer, inpatients, potential drug-drug interactions, risk factors, riskprediction model

## Abstract

**Objective:**

The potential drug-drug interactions (pDDIs) seriously affecting the prognosis of colorectal cancer (CRC) patients. This study aimed to identify the risk factors of pDDIs in hospitalized CRC patients and construct a risk prediction model to provide a reference for clinical rational drug use.

**Research design and methods:**

A retrospective cohort study was conducted, enrolling 2,868 patients from a tertiary hospital. Medscape was used to assess pDDIs, and a risk prediction model was constructed based on independent risk factors.

**Results:**

A total of 1,790 (62.41%) patients experienced at least one pDDIs, with 1,458 (50.84%) cases of severe pDDIs. The number of drug varieties, hypoalbuminemia, and treatment were independent risk factors. The area under the receiver operating characteristic curve (AUC) of the model in the training and validation sets were 0.826 and 0.824, respectively. The calibration curve showed a good agreement between the predicted probability and the actual occurrence probability. Decision curve analysis (DCA) demonstrated that the model had a positive net clinical benefit within a wide range of 10%–90%.

**Conclusion:**

The constructed model has good predictive performance and can be used to identify high-risk patients with pDDIs in clinical practice, thereby improving the safety of drug use.

## 1 Introduction

Colorectal cancer (CRC) is one of the most common malignancies worldwide, with a global incidence and mortality rate ranking among the top three of all cancers, imposing a heavy burden on public health and social economy (Bray et al., 2024; Filho et al., 2025). With the advancement of medical technology, the treatment of CRC has evolved into a comprehensive model combining surgery, chemotherapy, radiotherapy, targeted therapy, and immunotherapy (Wang et al., 2025a; Vilz et al., 2024; Yoshino et al., 2023). However, most CRC inpatients are elderly and often accompanied by multiple chronic comorbidities such as hypertension, diabetes, and cardiovascular diseases, leading to complex medication regimens (Alqenae et al., 2023; Chu et al., 2025; Endalifer et al., 2025; Guo et al., 2025).

Potential drug-drug interactions (pDDIs) refer to the potential changes in the efficacy or toxicity of one drug caused by the simultaneous or sequential use of another drug, which may result in adverse drug reactions (ADRs), treatment failure, or even life-threatening events (Alkathiri et al., 2025; Demirkapu and Cavdar, 2024; Khanna et al., 2024; Meslamani and Abou Hajal, 2025). For CRC inpatients, the risk of pDDIs is significantly increased due to the long treatment cycle, diverse therapeutic drugs, and combined use of drugs for comorbidities. Previous studies have shown that the incidence of pDDIs in cancer patients ranges from 55.7% to 95.0%, and severe pDDIs can increase the length of hospital stay and the mortality rate (Pinto et al., 2023; Choudary et al., 2022; Wondm et al., 2023; Milovanovic and Pejcic, 2025; Ismail et al., 2020). However, there are few studies specifically focusing on the risk factors of pDDIs in CRC inpatients, and the existing studies have small sample sizes and lack reliable risk prediction models, making it difficult to provide effective guidance for clinical prevention and intervention of pDDIs.

Therefore, this study conducted a retrospective study to systematically analyze the risk factors of pDDIs in hospitalized CRC patients, construct a risk prediction model using logistic regression, and evaluate the performance of the model. The study is expected to provide a scientific basis for clinical pharmacists and physicians to identify high-risk patients with pDDIs, formulate individualized medication plans, and reduce the occurrence of ADRs, thereby improving the prognosis and quality of life of CRC inpatients.

## 2 Materials and methods

### 2.1 Study design and participants

A retrospective study was conducted, and the study population was hospitalized CRC patients admitted to the Oncology Department of the First Affiliated Hospital of Bengbu Medical University (A large-scale comprehensive teaching hospital in Anhui province, China), from January 1 to 31 October 2023. The inclusion criteria were: (1) Patients diagnosed with CRC; (2) The types of drugs used ≥2; (4) complete medical records, including detailed medication information and clinical data. The exclusion criteria were: (1) The types of drugs used <2; (2) patients with incomplete medical records or missing key data (such as medication records, laboratory test results).

**FIGURE 1:**
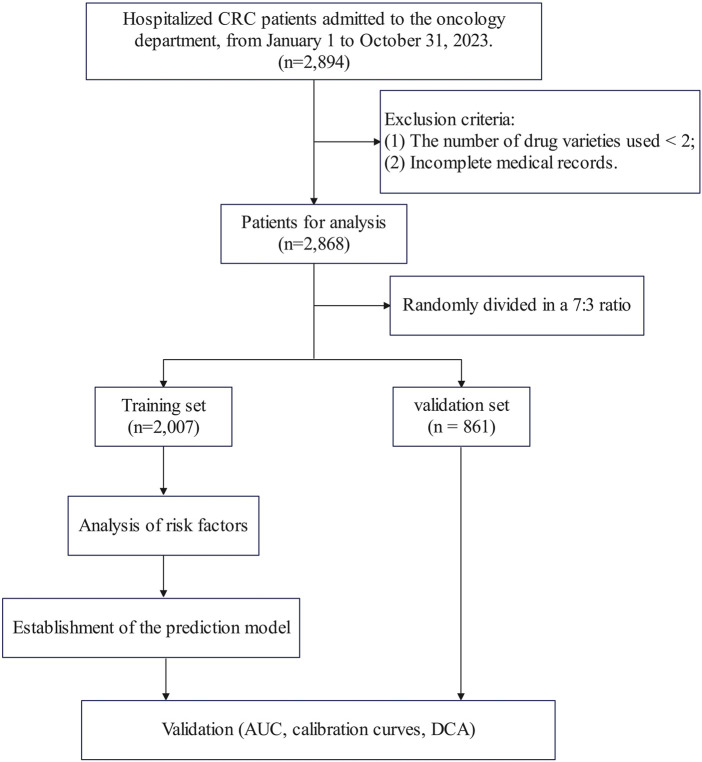
Flowchart of the study.

According to the above criteria, a total of 2,868 patients were included in this study. The patients were randomly divided into a training set (n = 2007, 70%) and a validation set (n = 861, 30%) in a 7:3 ratio. The flowchart was shown in Figure 1.

### 2.2 Ethical approval

The study protocol was performed in accordance with the Declaration of Helsinki and was approved by the ethics committee of the Bengbu Medical University (2024024), and the requirement for informed consent was waived due to there was no contact with the patient and the patient’s information was processed anonymously.

### 2.3 Data collection

A standardized data collection form was designed to extract data from the electronic medical records system, including the following aspects: age, gender, length of hospital stay, number of medication types, tumor metastasis status, and types of comorbidities (hypertension, coronary heart disease, diabetes, anemia, gastritis, renal insufficiency, hepatic insufficiency, thrombosis, cerebral infarction, intestinal obstruction, hypoproteinemia, etc.).

### 2.4 Assessment of pDDIs

Medscape, a widely used and authoritative drug interaction database, was used to assess pDDIs. According to the severity classification, pDDIs were divided into four types: “Contraindicated”, “Serious Use Alternative”, “Monitor Close”, “Minor”. In this study, patients with “Contraindicated” or “Serious Use Alternative” pDDIs were defined as the “Serious pDDIs group”.

The assessment of pDDIs was independently completed by two pharmacists. In case of disagreement, a third pharmacist was invited for consultation to reach a consensus.

### 2.5 Statistical analysis

All statistical analysis were performed using R software (Version 4.3.1) and SPSS software (Version 26.0). The statistical significance level was set at α = 0.05 (two-tailed).

Descriptive statistics: For measurement data, as they did not conform to a normal distribution, they were expressed as median (interquartile range) [M (Q1, Q3)], and the Mann-Whitney U test was used for comparison between groups. For count data, they were expressed as frequency (percentage) [n (%)], and the chi-square test or Fisher’s exact test was used for comparison between groups.

Screening of risk factors: First, univariate analysis was performed to compare the differences in variables between the serious pDDIs group and the non-serious pDDIs group. Then, variables with P < 0.1 in the univariate analysis were included in the multivariate logistic regression analysis to identify independent risk factors of serious pDDIs.

Construction and evaluation of the risk prediction model: Based on the independent risk factors identified by multivariate logistic regression and Akaike information criterion (AIC), a risk prediction model was constructed, and a nomogram was drawn using R software to visualize the model (R packages including pROC, ggplot2, rms, ResourceSelection, rmda). The receiver operating characteristic (ROC) curve, calibration curve, and decision curve analysis (DCA) are used to evaluate the discriminative ability, calibration ability, and clinical utility of the model, respectively (Xie et al., 2025).

## 3 Results

### 3.1 Baseline characteristics

A total of 2,868 hospitalized CRC patients were included in this study, including 1,701 males (59.31%) and 1,167 females (40.69%). The patients were randomly divided into a training set (n = 2,007) and a validation set (n = 861) at a ratio of 7:3. Comparison of clinical characteristics between the training and validation sets showed no statistically significant differences (P > 0.05), as detailed in Table 1.

**TABLE 1:** Baseline characteristics of patients in the training and validation sets.

Characteristics	Training set (n = 2,007)	Validation set (n = 861)	*P* value
Age, years	60 (54, 69)	60 (54, 69)	0.544
Gender, n (%)	​	​	0.143
Female	799 (27.9%)	368 (12.8%)	​
Male	1,208 (42.1%)	493 (17.2%)	​
Length of hospital stay, days	4 (3, 5)	4 (3, 5)	0.675
Number of drug varieties	12 (9, 14)	12 (9, 14)	0.909
Hypertension, n (%)	​	​	0.157
No	1,557 (54.3%)	647 (22.6%)	​
Yes	450 (15.7%)	214 (7.5%)	​
Coronary heart disease, n (%)	​	​	0.924
No	1971 (68.7%)	846 (29.5%)	​
Yes	36 (1.3%)	15 (0.5%)	​
Diabetes, n (%)	​	​	0.902
No	1,773 (61.8%)	762 (26.6%)	​
Yes	234 (8.2%)	99 (3.5%)	​
Anemia, n (%)	​	​	0.562
No	1,879 (65.5%)	811 (28.3%)	​
Yes	128 (4.5%)	50 (1.7%)	​
Gastritis, n (%)	​	​	0.646
No	1,968 (68.6%)	842 (29.4%)	​
Yes	39 (1.4%)	19 (0.7%)	​
Renal insufficiency, n (%)	​	​	0.778
No	1,991 (69.4%)	855 (29.8%)	​
Yes	16 (0.6%)	6 (0.2%)	​
Hepatic insufficiency, n (%)	​	​	0.121
No	1,938 (67.6%)	821 (28.6%)	​
Yes	69 (2.4%)	40 (1.4%)	​
Thrombosis, n (%)	​	​	0.927
No	1,990 (69.4%)	854 (29.8%)	​
Yes	17 (0.6%)	7 (0.2%)	​
Cerebral infarction, n (%)	​	​	0.281
No	1,929 (67.3%)	820 (28.6%)	​
Yes	78 (2.7%)	41 (1.4%)	​
Intestinal obstruction, n (%)	​	​	0.505
No	1987 (69.3%)	850 (29.6%)	​
Yes	20 (0.7%)	11 (0.4%)	​
Hypoalbuminemia, n (%)	​	​	0.736
No	1,933 (67.4%)	827 (28.8%)	​
Yes	74 (2.6%)	34 (1.2%)	​
Metastasis, n (%)	​	​	0.456
No	916 (31.9%)	406 (14.2%)	​
Yes	1,091 (38%)	455 (15.9%)	​
Treatment, n (%)	​	​	0.391
FOLFOX regimen	151 (5.3%)	77 (2.7%)	​
FOLFIRI regimen	155 (5.4%)	66 (2.3%)	​
CapeOx regimen	670 (23.4%)	270 (9.4%)	​
Targeted therapy	715 (24.9%)	295 (10.3%)	​
Others	316 (11%)	153 (5.3%)	​
Serious pDDIs, n (%)	​	​	0.383
No	976 (34%)	434 (15.1%)	​
Yes	1,031 (35.9%)	427 (14.9%)	​

Among them, a total of 3,647 pDDIs were identified, and 1,790 patients (62.41%) were found to have experienced at least one pDDIs. Based on severity classification, the numbers of pDDIs categorized as “Contraindicated”, “Serious - Use Alternative”, “Monitor Closely”, and “Minor” were 5, 1,749, 1,876, and 17, respectively. A total of 1,458 patients were divided into the severe pDDIs group, accounting for 50.84%.

### 3.2 Univariate and multivariate analysis of risk factors for pDDIs

The occurrence of severe pDDIs was set as the outcome variable. Univariate logistic regression analysis was first performed for each influencing factor, the variables with p < 0.1 were then included in a multivariate logistic regression analysis. The results are presented in Table 2. The analysis revealed that the number of drug varieties, hypoalbuminemia, and treatment were risk factors for the occurrence of severe pDDIs.

**TABLE 2:** Univariate and multivariate logistic regression analysis of factors for severe pDDIs.

Characteristics	Total (N)	Univariate analysis		Multivariate analysis	
		OR (95% CI)	*P* value	OR (95% CI)	*P* value
Age, years	2,007	0.992 (0.985–1.000)	0.057	1.003 (0.992–1.013)	0.613
Gender					
Female	799	Reference	​	​	​
Male	1,208	0.955 (0.798–1.142)	0.612	​	​
Length of hospital stay, days	2,007	1.002 (0.967–1.037)	0.921	​	​
Number of drug varieties	2,007	1.108 (1.083–1.133)	<0.001	1.097 (1.068–1.128)	<0.001
Hypertension					
No	1,557	Reference	​	​	​
Yes	450	0.880 (0.713–1.085)	0.232	​	​
Coronary heart disease					
No	1,971	Reference	​	​	​
Yes	36	1.498 (0.762–2.945)	0.241	​	​
Diabetes mellitus					
No	1773	Reference	​	​	​
Yes	234	1.097 (0.835–1.442)	0.505	​	​
Anemia					
No	1,879	Reference	​	Reference	​
Yes	128	0.608 (0.422–0.875)	0.007	0.715 (0.433–1.180)	0.189
Gastritis					
No	1,968	Reference	​	Reference	​
Yes	39	0.414 (0.208–0.822)	0.012	0.535 (0.235–1.220)	0.137
Renal insufficiency					
No	1,991	Reference	​	Reference	​
Yes	16	0.216 (0.061–0.761)	0.017	0.223 (0.047–1.063)	0.060
Hepatic insufficiency					
No	1,938	Reference	​	​	​
Yes	69	0.863 (0.534–1.396)	0.549	​	​
Thrombosis					
No	1,990	Reference	​	​	​
Yes	17	1.743 (0.642–4.731)	0.275	​	​
Cerebral infarction					
No	1,929	Reference	​	Reference	​
Yes	78	0.648 (0.409–1.026)	0.064	1.189 (0.663–2.131)	0.561
Intestinal obstruction					
No	1,987	Reference	​	Reference	​
Yes	20	0.234 (0.078–0.701)	0.010	0.392 (0.119–1.289)	0.123
Hypoalbuminemia					
No	1,933	Reference	​	Reference	​
Yes	74	0.174 (0.093–0.324)	<0.001	0.240 (0.113–0.510)	<0.001
Metastasis					
No	916	Reference	​	Reference	​
Yes	1,091	0.579 (0.485–0.692)	<0.001	0.989 (0.767–1.276)	0.933
Treatment					
FOLFOX regimen	151	Reference	​	Reference	​
FOLFIRI regimen	155	3.204 (1.581–6.496)	0.001	3.473 (1.691–7.132)	<0.001
CapeOx regimen	670	1.037 (0.672–1.598)	0.871	1.247 (0.799–1.947)	0.332
Targeted therapy	715	0.095 (0.062–0.146)	<0.001	0.125 (0.080–0.197)	<0.001
Others	316	0.051 (0.031–0.083)	<0.001	0.069 (0.042–0.116)	<0.001

### 3.3 Establishment of the risk prediction model

**FIGURE 2:**
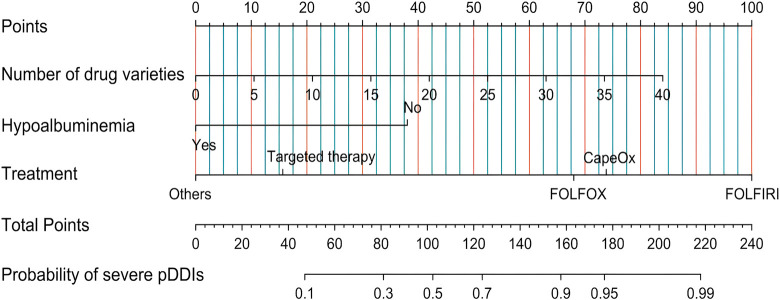
Nomogram for predicting the risk of severe pDDIs in hospitalized CRC patients. Note: For each patient, find the corresponding position of each risk factor on the upper axis, draw a vertical line downward to the “Points” axis to obtain the score of the factor, sum the scores of all factors to get the total score, and then draw a vertical line upward from the “Total Points” axis to the “Probability of severe pDDIs” axis to obtain the predicted probability of severe pDDIs.

Based on the independent risk factors identified through multivariate logistic regression analysis and AIC, a nomogram model was constructed to predict the occurrence of severe pDDIs in hospitalized CRC patients (Figure 2).

### 3.4 Validation of the predictive nomogram

The performance of the nomogram model was evaluated in the training set and the validation set, respectively.

**FIGURE 3:**
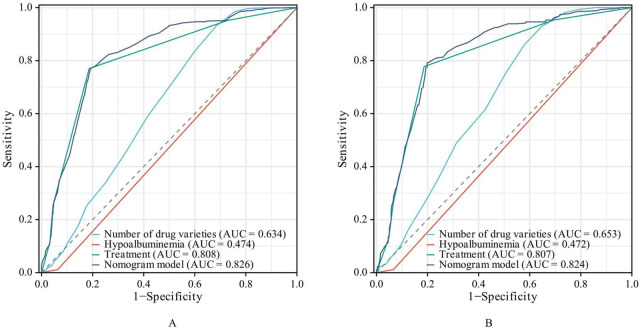
ROC Curves of the risk prediction model. (**(A)** training set. **(B)** validation set).

The AUC of the established nomogram model is larger than when the three influencing factors was applied separately, and the AUC of the nomogram model in the training and validation sets were 0.826 (95% CI: 0.807–0.844) and 0.824 (95% CI: 0.795–0.853), respectively, indicating favorable discriminative ability (Figure 3).

**FIGURE 4:**
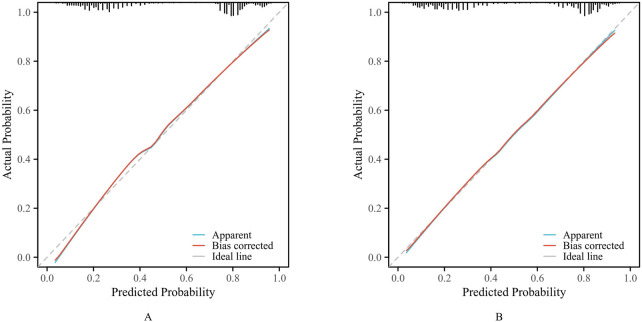
The calibration curves. (**(A)** training set. **(B)** validation set).

The calibration curve (Figure 4) showed that the predicted probability of pDDIs was in good agreement with the actual occurrence probability in both the training set and the validation set.

**FIGURE 5:**
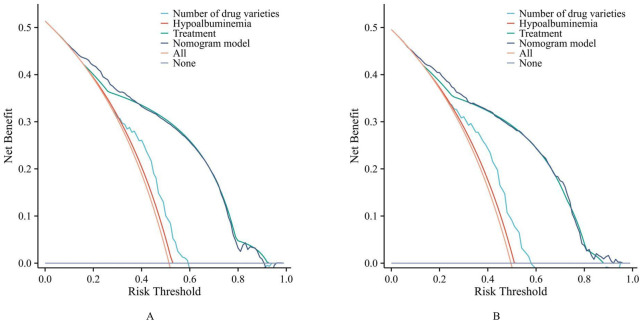
The decision curve analysis (DCA). (**(A)** training set. **(B)** validation set).

DCA (Figure 5) showed that when the threshold probability of pDDIs was between 10% and 90%, the net benefit of the model was higher than that of the “treat all” or “treat none” strategies in both the training set and the validation set, indicating that the model had good clinical utility.

## 4 Discussion

This retrospective cohort study systematically analyzed the risk factors of pDDIs in 2,868 hospitalized CRC patients and constructed a risk prediction model based on the identified independent risk factors. The results showed that the incidence of pDDIs in hospitalized CRC patients was 62.41%, which was consistent with the incidence of pDDIs in cancer inpatients reported in previous studies (55.7%–95.0%) (Choudary et al., 2022; Wondm et al., 2023; Milovanovic and Pejcic, 2025; Ismail et al., 2020). In our study, severe pDDIs accounted for 50.84%, indicating that pDDIs are a common and serious problem in hospitalized CRC patients, which requires high attention from clinical medical staff. However, to the best of our knowledge, no previous studies have investigated the prediction of pDDIs risks in hospitalized CRC patients. Based on real-world evidence, this study developed a risk prediction model for PDDIs in this population, which is expected to enhance the safety of treatment.

Based on univariate and multivariate logistic regression analyses, the independent risk factors ultimately identified in this study included the number of medication types, hypoalbuminemia, and treatment plans. Unlike previous studies (Saeed et al., 2025; Ramasubbu et al., 2025), age was not considered a risk factor for pDDIs in this study, possibly due to differences in study design, disease populations and regions.

Multiple studies have shown that the number of drug varieties is closely related to pDDIs, as the probability of pharmacokinetic and pharmacodynamic interactions increases combinatorially with the number of drugs administered. In this study, the OR for the number of drug varieties was 1.097 (95% CI: 1.068–1.128), suggesting that for each additional drug variety, the risk of pDDI increased by 9.7%. Furthermore, the specific treatment regimen employed also emerged as a critical determinant, which may be attributed to differences in metabolic pathways and adverse reactions, ultimately leading to variations in pDDIs (Wu et al., 2025; Peshin et al., 2025). For instance, in this study, the risk of pDDI was 3.47 times higher with the FOLFIRI regimen (Fluorouracil, Calcium Folinate, Irinotecan) compared to the FOLFOX regimen (Fluorouracil, Calcium Folinate, Oxaliplatin). This difference can be attributed to the complex metabolic pathways of irinotecan (involving carboxylesterases, CYP3A4, UGT1A1, etc.), which make it more susceptible to the effects of concomitant medications than oxaliplatin, which is primarily metabolized through non-enzymatic pathways (Zhu et al., 2023; Lentz et al., 2005; Liang et al., 2025).

Notably, hypoalbuminemia appears to be a protective factor against pDDIs, with patients in the hypoalbuminemia group having only 0.24 times the risk of pDDIs compared to those with normal albumin levels. In hypoalbuminemia, reduced plasma protein binding of drugs leads to increased free drug concentrations, significantly raising the risk of toxicity (Alvarez-Elias et al., 2017; Yang J. et al., 2025). Previous clinical studies have primarily focused on the relationship between hypoalbuminemia and clinical efficacy or adverse reactions (Lin et al., 2023; Wang et al., 2023; Liang et al., 2023; Tang et al., 2025). However, unlike earlier research, the clinical outcome in this study was the risk of pDDIs. The lower risk observed in the hypoalbuminemia group may be attributed to more cautious treatment plans (Prudent selection of drug types, reduction in the number of medications, and lower dosages) adopted by clinicians for this patient population (Wang et al., 2025b; Tan et al., 2024). Therefore, hypoalbuminemia should not be regarded as a protective factor against pDDIs, it should be understood as a clinical characteristic associated with more prudent medication management in this condition. What’s more, although the probability of pDDI risk is low, the severity of adverse reactions in real-world settings may be more severe (Ngcobo, 2025; Idasiak-Piechocka et al., 2025; Ling et al., 2024).

The nomogram prediction model, features a simple and intuitive interface along with high accuracy, thereby aiding clinicians in making better clinical decisions, its application in scenarios such as disease diagnosis and prognosis analysis is becoming increasingly widespread (Xu et al., 2025; Xie et al., 2025; Yang Y. et al., 2025; Huang et al., 2025). Based on the identified independent risk factors (aa, bb), this study developed and validated a nomogram for predicting the risk of serious pDDIs in patients with colorectal cancer.

As shown in Figure 2, the ROC curve demonstrated that the AUC of the nomogram constructed by combining these three indicators was higher than that of any single indicator. The AUC values of the nomogram were 0.826 in the training set and 0.824 in the validation set, indicating that the nomogram has a strong ability to identify patients at potential risk of severe DDIs. Calibration curves for both the training and validation sets indicated good agreement between actual and predicted diagnoses. Furthermore, DCA showed that within the threshold probability range of 10%–90%, using the nomogram for assessing patients’ DDI risk yielded satisfactory net benefits, indicating favorable clinical utility of the model. The established nomogram model can assist clinicians in more conveniently and rapidly identifying patients’ DDI risk, enabling targeted early intervention and improving medication safety.

The risk prediction model constructed in this study can help clinical medical staff identify high-risk patients with pDDIs in a timely manner. For patients with a high predicted risk (such as a probability ≥0.5 in the nomogram), clinical medical staff can take the following intervention measures: (1) Conduct a comprehensive medication review, including reviewing the patient’s medication history, comorbidities, and laboratory test results, and discontinuing unnecessary drugs; (2) Select drugs with fewer interactions, and adjust the dosage or administration time of drugs if necessary; (3) Strengthen the monitoring of patients’ clinical symptoms and laboratory indicators (such as liver and kidney function, coagulation function, drug concentration), and detect and handle ADRs in a timely manner; (4) Strengthen the communication between clinical pharmacists and physicians, and provide professional medication guidance for patients. These measures are expected to reduce the incidence and severity of adverse reactions, thereby improving treatment safety. However, the effectiveness of this model requires further validation in clinical practice.

This study also has some limitations: (1) This is a retrospective study, and there may be selection bias and information bias, which may affect the accuracy of pDDI assessment; (2) The study only included CRC patients from one hospitals in Anhui province, and the study population may have regional limitations; (3) The study did not consider the dosage and duration of drug use, which may also affect the occurrence of pDDIs; (4) The study did not conduct a prospective validation of the model, and the long-term predictive value of the model needs to be further verified in future studies.

## 5 Conclusion

The number of medication types, hypoalbuminemia, and treatment plans were risk factors for severe pDDIs in hospitalized CRC patients. The risk prediction model constructed based on these factors has good discriminative ability, calibration ability, and clinical utility, can be used as a simple and effective tool to assess the risk of pDDIs in CRC patients. This study provides a scientific basis for the prevention and intervention of pDDIs in hospitalized CRC patients, and is of great significance for improving the safety of drug use.

## Data Availability

The original contributions presented in the study are included in the article/supplementary material, further inquiries can be directed to the corresponding author.
